# Plasma Antioxidant Capacity Is Related to Dietary Intake, Body Composition, and Stage of Reproductive Aging in Women

**DOI:** 10.3390/antiox13080940

**Published:** 2024-08-02

**Authors:** Alexandra Tijerina, Diego Fonseca, Carlos J. Aguilera-González, Michel Stéphane Heya, Nancy Martínez, Nydia Sánchez, Cristina Bouzas, Josep A. Tur, Rogelio Salas

**Affiliations:** 1 Faculty of Public Health and Nutrition, Autonomous University of Nuevo Leon, Monterrey 64460, Mexicodiefo_2409@hotmail.com (D.F.); nydia.sanchez@gmail.com (N.S.);; 2 Faculty of Biological Sciences, Autonomous University of Nuevo Leon, Monterrey 66455, Mexico; carlos.aguileragn@uanl.edu.mx; 3 Research Group on Community Nutrition and Oxidative Stress, University of Balearic Islands–IUNICS, 07122 Palma de Mallorca, Spain; 4 Health Institute of the Balearic Islands (IDISBA), 07120 Palma de Mallorca, Spain; 5 CIBER Physiopathology of Obesity and Nutrition (CIBEROBN), Institute of Health Carlos III (ISCIII), 28029 Madrid, Spain

**Keywords:** antioxidant capability, women, reproductive, menopause, body composition, dietary intake

## Abstract

Background: women aging is a normal process of life; however, hormonal changes create an imbalance between prooxidants and antioxidants and could be measured as the antioxidant capability (AC) of an organism. Objective: to find the association between plasma AC levels, dietary intakes, and body composition in 18–64-year-old women living in the northeast of Mexico. Methods: A total of *n* = 514 women (18–64 years old) were grouped according to STRAW criteria as reproductive, menopausal transition, and postmenopausal. Anthropometrics, body mass index (BMI), weight–hip ratio (WHR), and weight–height ratio WHtR were determined, and percentage of body fat was analyzed by bioelectrical impedance. Dietary intake of macronutrients and vitamins A, E, and C were analyzed by a 3-day food recall. The AC status in plasma was analyzed by the ORAC_FL_ assay. Results: Plasma AC levels were higher in postmenopausal women (815 µmol TE/L), and menopausal transition women (806 µmol TE/L) than in reproductive women (633 µmol TE/L). BMI was overweight (>25 kg/m^2^) in all three groups. WHtR and WHR are above the healthy limit of 0.5 and 0.8, respectively for both menopausal transition and postmenopausal women. In reproductive women, negative relationships were calculated between plasma AC and age (*Rho* = −0.250, *p* = 0.007), BMI (*Rho* = −0.473, *p* < 0.001), WHtR (*Rho* = −0.563, *p* < 0.001), WHR (*Rho* = −0.499, *p* < 0.001), and % body fat (*Rho* = −0.396, *p* < 0.001). A negative association was determined between plasma AC and WHtR in reproductive women (*B* = −2.718, *p* = 0.026). No association resulted for those in menopausal transition, and a positive association was obtained between plasma AC and protein (*B* = 0.001, *p* = 0.024) and vitamin E (*B* = 0.003, *p* = 0.013) intakes in postmenopausal women. Conclusions: the antioxidant capability (AC) in plasma was lower in reproductive women, and anthropometric parameters marking decreased physical fitness were associated with decreased AC.

## 1. Introduction

Aging is a normal process of life for women; however, when reaching menopause, there is major change in hormonal levels, such as the decrease in estrogens (mainly estradiol) and anti-Müllerian hormone [1], thus causing generalized changes in metabolic function, muscle, and cardiovascular health [2]. These hormonal changes are of concern due to their association with chronic degenerative diseases [3,4] creating a greater imbalance between prooxidants and antioxidants in the organism, which is known as oxidative stress. The main factors in perimenopausal women associated with an increased oxidative stress include aging, decreased estrogen production, genetics, diet, and obesity [5].

The oxidative stress theory of aging [6] predicted that, as an inevitable byproduct of metabolic activity, reactive oxygen species (ROS) are produced. If these are not completely neutralized, oxidative damage to proteins, DNA, and lipids may occur. Unrepaired damage accumulates and results in the typical aging phenotype. Over the years, numerous studies have documented the role of oxidative stress caused by ROS in the aging process of higher organisms. Several age-associated disease models suggest that ROS and oxidative stress modulate the incidence of age-related pathologies and that it can strongly influence the aging process and possibly lifespan [7]. Environmental stressors and xenobiotics may induce DNA mutations, contributing to a high increase in ROS production, and, therefore, causing exogenous oxidative stress [7,8]. However, considerable data now exist to challenge the embattled oxidative stress theory [9]. Despite the marginal association with longevity, a dramatic difference is seen in the progression of age-related diseases. Therefore, manipulation of ROS levels via antioxidants may provide a reasonable remedy to many of the age-related diseases and distress [7].

The antioxidant capability (AC) of an organism is the ability to protect against oxidative stress damage via endogenous and exogenous compounds at intracellular and extracellular levels. The AC was positively associated with dietary intake of exogenous antioxidants derived from diet such as vitamins E, A, and C, carotenes, selenium, zinc, polyphenols, lycopene, quercetin, folate, and lutein, among other compounds [10,11,12], thus allowing reduction in inflammation while decreasing estradiol levels, the main endogenous antioxidant for women at reproductive stage [3,13].

However, the expected AC levels at the different stages of reproductive aging in women is still controversial. Previous studies reported higher antioxidant capability (AC) in postmenopause than in reproductive stages among Mexican women, which is related to higher oxidative stress in postmenopausal than in reproductive women [5], and similar findings were reported for another study conducted with Mexican DF women [14]. Spanish women in late postmenopause showed higher AC levels than those in early postmenopause [15], and similar results were obtained among Brazilian postmenopausal women who showed higher AC levels, too [16]. However, Russian women showed decreased AC levels only in postmenopause versus reproductive and perimenopause [17]. In other studies, total AC decreased in postmenopause [18,19,20,21].

Regarding role of genetics on AC, it was reported that the Caucasian population of the Southeastern European region showed high AC, and then, a heightened protection in the occurrence of oxidative stress showed a high distribution of the polymorphisms rs4880, rs1799895, rs660339, and rs28665122, which express the antioxidant enzymes. Another AC polymorphism (rs109179) is highly distributed in all the European population, and the non-Caucasian population seems more protected due to the high expression of AC polymorphisms rs1050450 and rs1138272. Then, Caucasian inhabitants from the Southeastern region of Europe may have higher oxidative stress protection than the Caucasian Northern, Central, Northwestern, and Southwestern Europeans [22]. 

Diet and obesity were proposed as an explanation of ethnic differences reflecting several contrary findings in studies regarding oxidative stress and AC measures [23]. A relationship between AC levels, dietary intake, and anthropometric measures in young women [24], in women in postmenopausal stage [15], and in 50–64-year-old women [25] was reported. A negative effect on the prooxidant–antioxidant balance due to excess of body fat, high waist circumference, and high body mass index (BMI) was reported in young adults, both men and women [26], suggesting unhealthy nutritional status or body composition as increasing free radical levels and thus causing oxidation of lipids, proteins, and nucleic acids of the body [27].

Therefore, there is still limited and contradictory evidence for the association of plasma AC with different stages of reproductive aging in women: reproductive, menopausal transition, and postmenopausal, but also between AC and body composition, and with dietary intake. Therefore, the aim of this study was to assess the association between plasma AC levels and reproductive stages, dietary intake, and body composition in 18–64-year-old women living in the northeast of Mexico.

## 2. Materials and Methods

### 2.1. Design and Study Participants

A descriptive cross-sectional study carried out in 2012–2017 in a representative population of the 18–64-year-old women living in the metropolitan area of Monterrey, Nuevo Leon state, Mexico (*n* = 560,115), according to the Censo de Población 2010 del Instituto Nacional de Estadística y Geografía e Informática (INEGI, Aguascalientes, México).

The sample size for the study was calculated to demonstrate the primary objective (AC levels in three reproductive stages: reproductive, menopausal transition, and postmenopause) with a statistical power of 80% and accepting an alpha risk of 0.05 and a beta risk of 0.2 in a two-sided test, 100 subjects were necessary in each group to recognize as statistically significant a minimum difference of 1 unit between any pair of groups if 3 groups exist. The common deviation is assumed to be 1.4, and a drop-out rate of 10% was anticipated. Therefore, a final sample of 514 subjects was selected. Software used: http://www.imim.es/ofertadeserveis/software-public/granmo/Version7 12 April 2012.

Women were invited to participate via social media, flyers posted in medical centers, university, and public areas, and by direct invitation outside supermarkets and department stores, and voluntary response sampling was followed for the recruitment. Exclusion criteria were any disease, avoiding natural feeding, pregnancy or lactation, abandonment of the study, and incomplete data. 

The study followed the Declaration of Helsinki and was approved by the Ethics Committee of the Facultad de Salud Pública y Nutrición (Protocols ID: 13-FaSPyN-SA-29 and 15-FaSPyN-SA-11; 20 March 2015). Written informed consent was obtained from participants. 

Interview and measurements were performed at the Center for Research in Nutrition and Public Health of the Faculty of Public Health and Nutrition, Autonomous University of Nuevo León. 

### 2.2. Clinical History and Sociodemographic Factors

Clinical history of participants included date of birth and age, current smoking habit (yes: ≥1 cigarette/day; no: <1 cigarette/day), date of last menses, duration and changes in menstrual cycles according to STRAW + 10 Criteria [1], systolic and diastolic blood pressure, heart frequency, and medication taken. Women were classified into three stages: (1) reproductive, as regular cycles or not noticeable changes were present (*n* = 116); (2) menopausal transition, as altered cycles (≥7 days) or intervals of amenorrhea (≥60 days) were present (*n* = 188); and (3) postmenopausal, as the absence of menstrual cycle for ≥12 months (*n* = 210).

Interviews and measurements were arranged on the day 20 ± 2 of participants’ menstrual cycle, specifically for reproductive and menopausal transition women, considering day 1 as the first day of menses, as suggested by Kim et al. [28] to avoid hemoglobin variations due to menstrual cycle variations. 

Sociodemographic factors such as participants’ civil status (married or couple, and single, divorced or widow), educational level (primary or no studies, secondary, and university), and current occupation (not working, housewife, working, and retired or handicap) were also registered. 

### 2.3. Anthropometrics and Body Composition

Height was measured using a digital stadiometer (SECA 274, ±2.0 mm, Sistemas de medición y básculas médicas · SECA–Mexico, Ciudad de Mexico, Mexico), with the participant’s head in the Frankfurt plane; weight was measured using a scale (Seca 874, ±0.1 kg, Mexico). Body weight (kg) and percentage of visceral body fat (%) was determined by bioelectrical impedance analysis, Inbody A120, and Software Lookin’Body v. 120 (Microcaya, Bilbao, Spain; https://www.composicion-corporal-inbody.com/LookinBody.html, accessed on 30 July 2024), based on eight electrodes and two frequencies (20 kHz and 100 kHz). Body mass index (BMI) was determined by the formula BMI = weight (kg)/height^2^ (m^2^), classified as obese ≥30 kg/m^2^, overweight 25–29.9 kg/m^2^, or normal weight 18.5–24.9 kg/m^2^ [29,30]. Waist and hip circumferences were measured using a non-stretch measuring tape (SECA 201, ±0.1 cm, Mexico) at the midpoint between the last rib and the iliac crest. Waist-to-hip ratio (WHR) was calculated by WHR = waist circumference (cm)/hip circumference (cm). Waist-to-height ratio (WHtR) was determined by the formula WHtR = waist circumference (cm)/height (cm). 

### 2.4. Blood Samples and Antioxidant Capacity Assay

Venous blood samples were obtained from the antecubital vein in tubes with an anticoagulant (EDTA-K_2_) after 12 h overnight fasting, as established by the Norma Oficial Mexicana NOM-253-SSA1-2012 [31]. The tube was centrifuged at 3500 rpm for 12 min. Deproteinization was performed prior to preservation. Plasma was obtained and frozen at −80 °C until the assays were performed. There were no effects of thawing the samples from −80 °C.

The antioxidant capability was determined by the oxygen radical absorbance capacity assay (ORAC_FL_) in deproteinized plasma samples (0.5M perchloric acid (HClO_4_) (1:1, *v*/*v*), according to Ou et al. [32] using the Fluoroskan Ascent FL equipment (Thermo Fischer Scientific Inc., Waltham, MA, USA) with readings during 60 min at an excitation of 485 nm and an emission of 527 nm, following the method previously reported [33,34].

The oxygen radical absorbance capacity (ORAC_FL_) assay measures a fluorescent signal from a probe that is quenched in the presence of reactive oxygen species (ROS), providing a measure of total antioxidant capability in the protein free plasma, using the same peroxyl radical generator for both lipophilic and hydrophilic antioxidants [35]. ORAC_FL_ is the most suitable for measuring ROS in biological samples (plasma, organic tissues). The radical 2, 2′-Azobis(2-amidinopropane) dihydrochloride (AAPH) can react with fluorescein. The substance whose ROS are to be measured exerts a protective effect on fluorescein by delaying the drop in fluorescence. This allows the area under the curve to be calculated and the different molecules to be compared. It works with solutions buffered at pH = 7.2–7.4 since it is very sensitive to pH variation [35].

Working on current plasma samples, phosphate buffer (BF) (75 mM) was prepared at pH 7.4. The Trolox calibrator was prepared in BF (20 mM Trolox equivalents (TE)) and stored in aliquots at −20 °C. A fluorescein sodium stock (FSS) (200 nM) was prepared and kept at 4 °C in a dark bottle. The peroxyl radical initiator (AAPH, 2, 2′-azobis(2-amidinopropane) di-hydrochloride) was prepared just before use, at a concentration of 60 mM. Fluorescence values were obtained at the end of the measurement. The area was calculated under the curve (AUC) by integrating over time (0–60 min) following the formula AUC= 0.5 + ΣAi/A1 + 0.5(A60/A0), where A was the fluorescence value, A0 = at 0 min, and A60 = at 60 min [32,33]. The AUC of the Trolox sample and calibrator were plotted on their concentration (μmol). From the AUC graph, the analysis was carried out through linear regression using the formula y = mx + b to obtain the slope m. The ORAC_FL_ value was obtained by dividing the slope of the sample by the Trolox Caliper pendant. The antioxidant capability (ORAC_FL_ value) was reported in plasma samples as Trolox equivalents (TE) per liter (L) of plasma: µmol TE/L.

### 2.5. Dietary Assessment

Food and beverage consumption was written down by participants using 3-day food recalls, two weekdays and one weekend day, on the week before participants’ interview and measurements. None of the women interviewed took dietary supplements.

Energy (kcal/d), protein intake (g/d), fat intake (g/d), carbohydrate intake (g/d), fiber intake/g/d), and vitamins A (µg RAE/d), E (mg/d), and C (mg/d) were analyzed using the software Food Processor^®^ version 15.0 (ESHA Research, Salem, OR, USA). Daily intakes of macro- and micronutrients were presented as raw intakes and adjusted for energy intake (intake per 1000 kcal) according to the nutrient density method [36].

### 2.6. Statistics

Statistical analyses were performed using IBM SPSS^®^ Statistics software (v.25, SPSS Inc., Chicago, IL, USA) and level of significance was set at *p* < 0.05.

Data were analyzed for normality by Kolmogorov–Smirnov test. Descriptive statistics are presented for participants’ characteristics. Median and quartiles (Q1, Q3) were determined for numerical variables and differences among stages (reproductive, menopausal transition, and postmenopause) were analyzed by Kruskal–Wallis test and Bonferroni post hoc was applied to interpret differences among stages. Frequency (*n*) and percentage of response (%) were calculated for categorical variables, and differences among stages were determined by Chi-squared test (*Χ*^2^).

A Spearman correlation analysis was performed to understand the relationship between the ORAC_FL_ value, age, body composition, and dietetic measures. A multiple linear regression model was proposed to assess the association between plasma ORAC_FL_ value (dependent) and body composition measures and dietetic variables (independent) for the three stages of reproductive aging. The model was adjusted for age (yr), BMI (kg/m^2^), energy intake (kcal), and smoking habit (yes/no). 

## 3. Results

Table 1 shows the sociodemographic characteristics of the sample. Except for age, there were no differences between the three reproductive stages. The smoking habit was reported by 2.8% of reproductive women, 8.5% of menopausal transition, and 8.2% of postmenopausal women (*p =* 0.079).

Table 2 shows clinical and nutritional characteristics from participants, showing differences between groups (*p* < 0.001), except for protein (*p* = 0.438) and fiber intake (0.183), as well as for systolic and diastolic blood pressure, heart frequency, and medication. Plasma ORAC_FL_ values resulted in higher capacity in postmenopausal women (815 µmol TE/L), and menopausal transition women (806 µmol TE/L), than in reproductive women (633 µmol TE/L). BMI, intakes of energy, fat, protein, carbohydrates, and vitamin E were not different between menopausal transition vs. postmenopausal women. Reproductive women showed the lowest energy intakes (1566 kcal/d) than the other groups (*p* < 0.001).

BMI was overweight (>25 kg/m^2^) in all three groups. WHtR and WHR were above the healthy limit of 0.5 and 0.8, respectively, for both menopausal transition and postmenopausal women. Reproductive women were below the limits 0.47 for WHtR and 0.74 for WHR. The median values of body fat resulted in >30% in all groups (30.8 to 42.7%); menopausal and postmenopausal women showed median body fat above 40%. Women at the three reproductive stages showed similar data of blood pressure, and heart frequency. Medication intake was lower at reproductive stage, but higher at postmenopause, without significative differences between them.

The correlation analysis of plasma AC was conducted between age and other study outcomes (Table 3). WHtR and WHR were correlated in all three groups (*p* < 0.05). However, correlations showed a negative relationship in reproductive women in age (*Rho* = −0.250, *p* = 0.007), BMI (*Rho* = −0.473, *p* < 0.001), WHtR (*Rho* = −0.563, *p* < 0.001), WHR (*Rho* = −0.499, *p* < 0.001), and percent of body fat (*Rho* = −0.396, *p* < 0.001) and a positive relationship with plasma AC in menopausal transition and postmenopausal women. WHtR showed a higher correlation coefficient with plasma AC in menopausal transition (*Rho* = 0.204, *p* = 0.005) and postmenopause (*Rho* = 0.196, *p* = 0.004). Protein intake in postmenopausal women was also correlated with plasma AC (*Rho* = 0.134, *p* = 0.049). No other differences were reported.

A multiple regression model (Table 4) was performed to assess the association of plasma AC with nutritional variables in the study groups. A negative association was determined between plasma AC and WHtR in reproductive women (*B* = −2.718, *p* = 0.026). No association resulted for those in menopausal transition, and a positive association was obtained between plasma AC and protein (*B* = 0.001, *p* = 0.024) and vitamin E (*B* = 0.003, *p* = 0.013) intakes in postmenopausal women. 

## 4. Discussion

The main findings of the current study are that plasma antioxidant capability (AC) was lower in reproductive women and higher in postmenopausal women, and anthropometric parameters marking decreased physical fitness were associated with decreased AC levels. Protein and vitamin E intake was slightly correlated with AC in postmenopausal women.

The natural aging of women is a process in which estrogen levels will decrease, thus suggesting a decrease in antioxidant protection of the organism. Scientific evidence showed that women in postmenopause were more susceptible to oxidative stress and had a higher antioxidant capacity than reproductive women [16]. In Mexican City women, menopause was the main risk factor for oxidative stress, which was attributed to depletion of estrogen in this reproductive stage [14]. Despite the detrimental levels of estrogen, significantly higher AC levels were found in postmenopausal women but without significant differences between reproductive stages [14].

The measurement of total AC reflects the antioxidative status of an organism [37]. In the current study, plasma AC was lower in reproductive women (633 µmol TE/L) than in menopausal transition (806 µmol TE/L) and higher in postmenopausal women (815 µmol TE/L). There were no differences between the menopausal transition and postmenopausal stages. These findings are similar to the previous report about Mexican DF women [14] with AC levels 14% higher in postmenopausal women than those in the reproductive stage, or in Brazilian women with around 20% higher AC [16], and our results with 22% higher AC levels at this reproductive stage. This evidence suggests that the organism of menopausal women has already compensated for the loss of estrogens as an adaptive process, increasing the production of antioxidants [16]; thus, the AC at this stage of aging could be augmented as a natural response to neutralize free radicals. 

Statistical differences in several nutritional variables among stages (*p* < 0.001) supports each group’s independence. In general, reproductive stage women were younger and had lower anthropometric ratios (WHtR and WHR) and percentage of body fat; although, a percentage of body fat above 30% and a BMI of overweight (25.5 kg/m^2^) were also determined at this stage when participants were young (18–35 years). Ratios determined herein are of concern especially at this young stage, as previous studies reported an increase above 5% in BMI during 20 years of follow-up at menopausal–postmenopausal stages, and increments were due to increase in body weight or decrease in stature [38]; while increases in central body fat were also reported even in nonobese reproductive women through menopausal transition mainly without cardiometabolic risks [39]. Results in body composition are as expected for menopausal transition and postmenopausal women, in which visceral fat distribution occurs, as WHR was above 0.85 [40] and WHtR above 0.5 [41,42], in addition to the percentage of body fat being above 40%, demonstrating cardiovascular risk.

Higher intakes of energy, fat, and vitamins E, C, and A were found in menopausal transition and postmenopausal women but were lower for protein and carbohydrate intakes compared to those intakes of reproductive women. Duval et al. [43] also reported higher intakes of dietary fat in postmenopausal women, and menopausal transition was seen as a stage in which women tended to have a decrease in energy intake and an increased desire to eat. Intakes of antioxidant vitamins were below the Mexican daily recommended intakes for vitamin E (13 mg/d) in all stages (<8 mg/d), vitamin C was not adequate in reproductive women (<43 vs. 75 mg/d), and neither was vitamin A in the same stage (<278 vs. 570 μg RAE/d). A possibility with these low intakes may be because higher BMI is related to greater under-reporting when using self-reported dietary assessment tools [44] such as the 3-day food records.

In the current study, plasma AC was significantly related with anthropometric measures in all groups of women, but quite selective with intakes. Although consumption of antioxidant vitamins is considered protective against free radicals and oxidative stress in direct and indirect mechanisms [45], women’s low intakes may be a possible reason for the lack of statistical relationship with plasma AC levels. Negative relationships were determined in the reproductive stage (*Rho* −0.563 to −0.250), like Hermsdorff et al. [46], who reported a negative relationship between AC levels and waist circumference in young adults from Brazil and Spain. A suggested explanation for this finding is the dependence of an endogenous antioxidant, superoxide dismutase (SOD), on body weight. A greater body weight or BMI was related with increased protein carbonyl levels, and with decreased antioxidant capability levels due to lower SOD activity, which agrees with a previous meta-analysis that showed a decrease in total AC and an increase in markers of oxidative stress in obesity [5,47]. On the contrary, Jakubiak et al. [48] reported a positive correlation of AC with obesity and metabolic disorders in young Polish women (18–36 years). Menopausal transition and postmenopausal women resulted in positive relationships between plasma AC and BMI, WHtR, WHR, and percentage of body fat (menopausal transition), and with WHtR, WHR, and protein intake, but not BMI (postmenopausal). Similarly, moderate direct relationships between AC and age (*r* = 0.22, *p* = 0.039), BMI (*r* = 0.43, *p* < 0.001), and waist circumference (*r* = 0.42, *p* < 0.001) in healthy Lithuanian women (50–64 years) were demonstrated [25]. On the contrary, a study in postmenopausal Spanish women (44–76 years) found an inverse relationship between AC and weight (*r* = −0.273, *p* = 0.016) and body fat mass (*r* = −0.224, *p* = 0.049) [15]. However, previous experiences and those from the current study are not strictly comparable, since the ages of the Polish and Spanish women are not the same, with the Polish being younger (18–36 years), and the Spanish, older (44–76 years). Current findings in Mexicans were obtained at the age of 18–64 years. Further findings should be obtained after working at the same age. 

Another suggested explanation contributing to these contradictory findings could be the influence of several factors such as ethnic and genetic differences among study groups [23], sample sizes used [49], and the assay used to assess the antioxidant capability. Regarding the ethnicity, it was reported that African American adults showed significantly higher total AC, SOD, and protein carbonyl levels than Caucasian American adults [50]; however, Russian climacteric women showed low AC levels, which was attributed to a decrease in postmenopausal estrogen levels linked to their ethnicity [17] but also to the different analysis method used [5]. The same could be said about the current recruited Mexican women. However, they did not show ethnic differences between them, when they were clearly a mixed ethnical origin from Mexican native and Spanish origin. Further findings should be obtained after determining relationships between AC and genetics. 

Regarding the method used, in the current study, the ORAC_FL_ assay was selected since it is photo-stable, sensitive and specific, and has been widely used for assessing the antioxidant capability in biological and food samples [35,51]. Although no markers of oxidative stress were measured in this research, the analysis of the antioxidant capacity in plasma by the ORAC_FL_ assay reflects the endogenous and exogenous antioxidant status [32,35,51] of the organism that provides protection against free radical damage. 

A statistical approach using a multiple linear regression model was selected to adjust for several independent variables that could affect the study results, although no causal effect can be expressed due to the study design. After adjusting for age, BMI, energy intake, and smoking habit, the model for reproductive women was statistically significant only for waist-to-height ratio (WHtR). A decrease in 2.718 µmol TE/L in plasma antioxidant capacity was demonstrated with an increase in 0.01 unit in WHtR. This result is consistent with the literature as this anthropometric ratio has been associated to visceral fat, as increasing proinflammatory activity [52] and decreased anti-inflammatory activity, thus, decreasing the plasma AC value. 

In the stage of menopausal transition, there was no association observed between plasma AC and the analyzed factors. Coefficients of association in the model for postmenopausal women showed that an increase of protein intake in 1 g resulted in an increase in 0.001 µmol TE/L in plasma AC and an increase in vitamin E intake in 1 mg resulted in an increase in plasma AC of 0.003 µmol TE/L. In a previous study, there was no association reported between the antioxidant capacity and vitamin E intake in postmenopausal women from Iran [53]. Although association resulted in low coefficients in postmenopausal women, findings support the evidence on the fact that AC levels of an organism may be directly affected by women’ dietary antioxidants intake [54]; thus, suggesting adequate dietetic consumption for macro- and micronutrients and varying the food consumed, especially those foods that could increase the antioxidant capacity in the organism such as fruits and vegetables are beneficial [55,56]. Another consideration may be the use of a dietetic antioxidant capacity value (by using databases for the calculation derived from known antioxidant assays), as dietary components should be seen as having a synergistic antioxidant effect, rather than individual effects [49,57], although drawbacks have occurred due to lack of direct physiological effects [58]. It was suggested that future randomized clinical trials should evaluate the efficacy of interventions, like diet, supplementation, hormonal replacement, lifestyle, and others, to improve antioxidant defenses in postmenopausal women [5,59].

## 5. Strengths and Limitations

The main strength of this current research is that the plasma antioxidant capacity is associated with the stage of reproductive aging. Moreover, this is the first study where AC in the menopausal transition stage has been assessed. The main limitation of the current study is the cross-sectional design that avoids establishing causal interferences, and prospective analysis should be developed in future research. Another limitation is that the energy expenditure was not measured using the doubly labeled water method or other techniques; despite this, in this study, the energy intake was calculated from the three 24 h recalls as is usual in this kind of study. Finally, the lack of control of some variables like estrogen or other hormone concentrations, markers of metabolic status, and antioxidant biomarkers is another limiting factor; so, the current findings should be interpreted with caution.

## 6. Conclusions

The current study findings suggest that the antioxidant capability (AC) in plasma is lower in reproductive women, and anthropometric parameters marking decreased physical fitness are associated with decreased AC. It was negatively affected by the weight-to-height ratio in reproductive women, while it was positively affected by protein and vitamin E intake in postmenopausal women. Further studies are suggested to support these study results.

## Figures and Tables

**Table 1:** Sociodemographic characteristics of the sample.

	Reproductive (*n* = 116)	Menopausal Transition (*n* = 188)	Postmenopause (*n* = 210)	*p*-Value
Age (years old) *	25 ± 3 ^a^	45 ± 4 ^a^	54 ± 3 ^a^	0.019
Civil status ^#^				0.106
Married or couple	82.8%	85.7% ^b^	69.0% ^b^	
Single, divorced or widow	17.1%	14.3%	31.0%	
Educational level ^#^				0.293
Primary or no studies	0.7%	5.1%	9.0%	
Secondary	56.9%	49.6%	46.9%	
University	42.4%	45.3%	44.1%	
Current occupation ^#^				0.301
Not working	3.6%	10.3%	8.6%	
Housewife	56.8%	37.3%	36.0%	
Working	41.3%	60.6%	50.1%	
Retired or handicap	1.9%	2.0%	14.0%	
Smoking status ^#^				0.079
Yes (≥1 cigarette/day)	2.8%	8.5%	8.2%	
No (<1 cigarette/day)	97.3%	91.6%	91.8%	

Categorical variables are shown as frequency (percentage). * Kruskal–Wallis test was used to observe differences among groups. ^a,b^ denotes difference among stages calculated by post hoc Bonferroni test. Significance if *p* < 0.05. ^#^ Differences by Chi-squared test.

**Table 2:** Clinical and nutritional characteristics of study participants grouped according to the three reproductive stages.

	Reproductive (*n* = 116)	Menopausal Transition (*n* = 188)	Postmenopause (*n* = 210)	*p*
Plasma antioxidant capability (µmol TE/L)	633 (424, 822) ^a^	806 (689, 909) ^b^	815 (724, 950) ^b^	<0.001
BMI (kg/m^2^)	25.5 (21.7, 30.6) ^a^	28.4 (24.6, 32.9) ^b^	28.8 (25.7, 33.5) ^b^	<0.001
Waist-to-height ratio (WHtR)	0.47 (0.42, 0.56) ^a^	0.57 (0.52, 0.62) ^b^	0.59 (0.54, 0.65) ^c^	<0.001
Waist-to-hip ratio (WHR)	0.74 (0.70, 0.81) ^a^	0.85 (0.80, 0.89) ^b^	0.87 (0.81, 0.91) ^c^	<0.001
Body fat (%)	30.8 (22.6, 39.0) ^a^	41.0 (36.8, 45.3) ^b^	42.7 (37.8, 47.2) ^c^	<0.001
Systolic Blood Pressure (mmHg)	108 (98, 117)	112 (101, 129)	116 (107, 129)	0.115
Diastolic Blood Pressure (mmHg)	70 (62, 77)	73 (63, 83)	72 (64, 81)	0.404
Heart frequency (bites/minute)	69 (60, 75)	70 (63, 77)	67 (61, 74)	0.232
Medication				
Hormone therapy (*n*, %)	5 (4.1%)	16 (8.5%)	29 (13.8%)	0.093
Hypocholesterolemics (*n*, %)	2 (1.7%)	8 (4.2%)	30 (14.3%)	0.155
Oral hypoglycemics (*n*, %)	2 (1.7%)	12 (6.4%)	33 (15.7%)	0.186
Antihypertensive agents (*n*, %)	6 (5.2%)	17 (9.0%)	34 (16.2%)	0.280
Thyroid medications (*n*, %)	0 (0.0%)	9 (4.8%)	30 (14.3%)	0.098
Analgesics (*n*, %)	10 (8.6%)	21 (11.2%)	39 (18.6%)	0.498
Others (*n*, %)	26 (22.4%)	46 (24.5%)	62 (29.5%)	0.120
Energy intake (kcal/d)	1566 (1240, 1805) ^a^	2593 (2151, 3329) ^b^	2571 (2123, 3139) ^b^	<0.001
Fat intake (g/1000 kcal) *	37.5 (31.9, 41.3) ^a^	40.7 (36.5, 44.6) ^b^	39.8 (35.0, 43.9) ^b^	<0.001
Protein intake (g/1000 kcal/) *	42.3 (37.3, 49.6)	41.7 (36.8, 47.1)	41.7 (36.9, 47.0)	0.438
Carbohydrates intake (g/1000 kcal) *	127.9 (114.1, 138.2) ^a^	121.8 (107.5, 133.1) ^b^	122.9 (111.8, 136.0) ^b^	0.018
Fiber intake (g/1000 kcal)	23.6 (18.8, 31.9)	37.0 (14.3, 1.1)	40.1 (15.6, 22.4)	0.483
Vitamin E intake (mg/1000 kcal) *	0.8 (0.5, 1.4) ^a^	2.7 (2.2, 3.4) ^b^	2.9 (2.3, 3.8) ^b^	<0.001
Vitamin C intake (mg/1000 kcal) *	27.1 (16.8, 44.0) ^a^	73.2 (48.1, 106.4) ^b^	85.8 (57.5, 126.9) ^c^	<0.001
Vitamin A intake (µg RAE/1000 kcal) *	194.4 (105.8, 292.7) ^a^	298.7 (210.4, 417.5) ^b^	341.3 (242.3, 470.2) ^c^	<0.001

Values of continuous variables are expressed as median (Q1, Q3). Categorical variables are shown as frequency (percentage). Kruskal–Wallis test was used to observe differences among groups. ^a,b,c^ denotes difference among stages calculated by post hoc Bonferroni test. Significance if *p* < 0.05. * Macro- and micronutrient intakes are shown as daily intake per 1000 kcal.

**Table 3:** Correlation between plasma antioxidant capability and age and nutritional outcomes in the three reproductive stages: reproductive, menopausal transition, and postmenopause ^a^.

	Reproductive (*n* = 116)		Menopausal Transition (*n* = 188)		Postmenopause (*n* = 210)	
	*Rho*	*p*	*Rho*	*p*	*Rho*	*p*
Age (yr)	−0.250	0.007	−0.035	0.631	−0.004	0.955
BMI (kg/m^2^)	−0.473	<0.001	0.154	0.035	0.133	0.054
Waist-to-height ratio (WHtR)	−0.563	<0.001	0.204	0.005	0.196	0.004
Waist-to-hip ratio (WHR)	−0.499	<0.001	0.188	0.010	0.173	0.012
Body fat (%)	−0.396	<0.001	0.164	0.025	0.042	0.542
Protein intake (g/1000 kcal)	−0.121	0.196	0.023	0.754	0.134	0.049
Fat intake (g/1000 kcal)	−0.140	0.134	−0.003	0.968	0.091	0.190
Carbohydrate intake (g/1000 kcal)	0.013	0.889	−0.021	0.770	0.008	0.914
Fiber intake (g/d)	0.014	0.808	−0.031	0.680	0.007	0.902
Vitamin E intake (mg/1000 kcal)	0.012	0.900	−0.127	0.081	−0.060	0.386
Vitamin C intake (mg/1000 kcal)	0.105	0.262	−0.046	0.527	0.032	0.642
Vitamin A intake (µg/1000 kcal)	0.040	0.671	−0.066	0.370	0.097	0.160
Smoking habit (Yes/No)	0.023	0.803	−0.043	0.557	0.071	0.307

^a^ Models for the three groups were adjusted for age, BMI, energy intake, and smoking habit.

**Table 4:** Multiple linear regression analysis to determine the association of plasma AC with nutritional variables in three groups of women: reproductive, menopausal transition, and postmenopause ^a^.

Variables	Reproductive (*n* = 116)		Menopausal Transition (*n* = 188)		Postmenopause (*n* = 210)	
	B (95% CI)	*p*	B (95% CI)	*p*	B (95% CI)	*p*
Waist-to-height ratio (WHtR)	−2.718 (−5.098, −0.0338)	0.026	0.351 (−0.361, 1.063)	0.332	0.587 (−0.473, 1.648)	0.276
Waist-to-hip ratio (WHR)	0.802 (−0.398, 2.002)	0.188	0.017 (−0.064, 0.098)	0.679	−0.147 (−0.768, 0.474)	0.642
Body fat (%)	0.004 (−0.005, 0.012)	0.390	0.002 (−0.005, 0.009)	0.654	−0.005 (−0.010, 0.001)	0.091
Protein intake (g/d)	0.001 (−0.002, 0.004)	0.460	0.000 (−0.002, 0.001)	0.485	0.001 (0.000, 0.002)	0.024
Fat intake (g/d)	−0.003 (−0.006, 0.001)	0.102	0.000 (−0.001, 0.002)	0.769	0.000 (−0.001, 0.001)	0.817
Carbohydrate intake (g/d)	0.001 (0.000, 0.002)	0.093	0.018 (0.000, 0.000)	0.903	0.000 (−0.001, 0.001)	0.169
Fiber intake (g/d)	0.002 (0.000, 0.003)	0.193	0.005 (0.001, 0.010)	0.803	0.001 (−0.001, 0.002)	0.359
Vitamin E intake (mg/d)	0.004 (0.008, 0.016)	0.497	0.000 (−0.008, 0.009)	0.966	0.003 (0.001, 0.012)	0.013
Vitamin C intake (mg/d)	0.000 (−0.001, 0.001)	0.553	−0.006 (0.000, 0.000)	0.956	0.000 (0.000, 0.000)	0.410
Vitamin A intake (µg/d)	0.000 (0.000, 0.000)	0.124	−0.002 (0.000, 0.000)	0.526	0.000 (0.000, 0.000)	0.248

^a^ Models for the three groups were adjusted for age, BMI, energy intake, and smoking habit.

## Data Availability

There are restrictions on the availability of data for this trial due to the signed consent agreements around data sharing, which only allow access to external researchers for studies following the project purposes. Requestors wishing to access the trial data used in this study can make a request to pep.tur@uib.es.
